# Flourishing alongside psychiatric symptoms: a qualitative study on dual-continuum mental health in university students

**DOI:** 10.3389/fpsyg.2026.1980855

**Published:** 2026-09-23

**Authors:** Ingrid Vargas-Huicochea, Rodrigo Camargo-Cabello, Silvia Aracely Tafoya, Jaqueline Cortés, Olga Marina Robelo-Zarza

**Affiliations:** 1 Department of Psychiatry and Mental Health, Faculty of Medicine, Universidad Nacional Autónoma de México (UNAM), Mexico City, Mexico; 2 Universidad de los Andes, Bogotá, Colombia

**Keywords:** ecological systems theory, flourishing, languishing, mental health continuum, professional identity formation, psychological safety, qualitative research, college students / univerisity students

## Abstract

**Background:**

University students’ mental health is typically assessed through a deficit-oriented lens that equates symptom absence with well-being. Keyes’ dual-continuum model challenges this assumption, yet qualitative evidence examining how students in active psychiatric treatment narrate the coexistence of distress and flourishing remains scarce, particularly outside high-income, English-speaking contexts.

**Methods:**

A qualitative descriptive-interpretive study was conducted with seven undergraduate students (six women, one man; ages 19–23) receiving outpatient psychiatric care at a university-affiliated clinic in Mexico City. Participants were recruited following a quantitative survey week; those who volunteered for further exploration were interviewed. Semi-structured interviews were triangulated with clinical records and analyzed using reflexive thematic analysis, integrating deductive categories from Keyes’ eleven well-being facets with inductively emergent themes.

**Results:**

Five cross-cutting themes emerged: (a) flourishing and psychiatric symptoms co-occurred instead of alternating; (b) the university functioned as an ambivalent microsystem, simultaneously protective and threatening; (c) vital interest operated as a motivational hinge linking emotional, psychological, and social well-being; (d) peer networks functionally replaced family-of-origin support; and (e) institutional disciplinary processes could profoundly compromise psychological safety in a critical illustrative case. Clinical records were consistent with students’ narratives of high flourishing alongside substantial psychiatric burden.

**Conclusion:**

Flourishing and psychiatric symptoms can coexist within the same individual and narrative. University mental health services should assess flourishing and symptom severity as complementary but distinct tasks, and attend to ecological and institutional factors, including psychological safety around disciplinary processes, that shape students’ mental health trajectories.

## 1 Introduction

Most mental disorders develop in the university years. This temporal focus is why students suffer more from untreated psychological distress than students in general, even as they are studying outside of college. The WHO World Mental Health International College Student initiative surveyed 13,000 first-year students in eight countries. More than one in three students had some level of diagnostic score for at least one common mental disorder in the last 12 months—predominantly major depressive disorder and generalized anxiety disorder (Auerbach et al., 2018). National epidemiological surveys in Mexico indicate that anxiety disorders, substance use, and major depressive disorder remain highly prevalent in the general population (Medina-Mora et al., 2003). University students bear more of the burden than we might expect, given the academic, social, and economic conditions to get a degree (Vargas-Huicochea et al., 2022). They also go to professional services less frequently than the rest of the population. Cost and stigma are part of the reason why. Students understand their own suffering before any external intervention is even attempted (Vargas-Huicochea et al., 2022).

The literature still correlates symptom absence with well-being. Keyes (2002, 2005) challenged this assumption. Mental illness and mental health are linked but on very different axes and not opposite poles of one continuum. Flourishing requires high levels across eleven dimensions of emotional, psychological, and social well-being. Languishing is the absence of such well-being, regardless of diagnosis. A person who has serious depression but is not diagnosed can still be very successful in purpose, relationships, and social contributions (Keyes, 2005, 2007). Community and student samples have also been found to show this pattern on the Mental Health Continuum Short Form (Lamers et al., 2011). What is missing from the evidence base is qualitative testimony from people who are not merely being checked for symptoms but who are actively engaged in psychiatric treatment—how this coexistence sounds to those living it.

An additional conceptual limitation is not health but where distress is assumed to occur. Most studies focus on the students: personality, coping styles, and meaning-making processes. The context is not often addressed. Bronfenbrenner (1979, 1994) has developed an ecological model to question this internal focus. Human development occurs inside nested systems. Microsystems are the environments where students are directly interacting with each other; in classrooms, peer groups, and families. Mesosystems link these microsystems together. Exosystems are also a factor that shape students indirectly: institutional policies, parental employment. A macrosystem of cultural norms defines all the other levels of the world. The question is then shifted from what a student feels to where in this layered structure that feeling comes from, persists, or gets resolved.

There are two more constructs to consider as every student in our study was preparing for a job (not just a university degree). Professional identity formation is the process in which students slowly learn a profession’s values and skills as they grow up, and develop them into part of the identity and not as individual skills (Cruess et al., 2014; Cruess et al., 2016). Psychological safety (a term borrowed from organizational research) is a common belief that an environment would tolerate people’s risks (error, need for help) without having them embarrassed or punished for it (Edmondson, 1999). Although originally conceptualized for organizational work teams, psychological safety is highly transferable to educational settings. In the context of university micro- and mesosystems, it provides a crucial lens for understanding how institutional structures—such as disciplinary tribunals and adjudicative processes—shape students’ willingness to seek help and their fundamental sense of belonging without fear of punitive reprisal. It has been a topic that has developed in clinical training in the last few years, with learners having a greater level of openness to share personal experiences (Dong et al., 2025). Both constructs emerge, in Bronfenbrenner’s terms, at mesosystem and exosystem levels, from institutional structures and not individual characteristics.

These pressures are especially strong in Mexico and in Latin America, with the outcome being a product of wider conditions that go beyond the university or the student themselves. The academic demands of Mexican professionals often come to be linked to family expectations of social mobility. First-generation students are affected the most and report a high degree of psychological distress (Vargas-Huicochea et al., 2022). Stigma surrounding help-seeking persists even among students training for health professions. A study of 305 Mexican medical students found that attitudinal and structural obstacles were more important than the availability of services (Quiñones-Robles et al., 2025). The reason is a regional deficit. In Latin America and the Caribbean, the World Health Organization estimates the mental health treatment gap is 77.9% and the median mental health expenditure is only 2.1% of regional health budgets (World Health Organization, 2025). The Pan American Health Organization’s High-Level Commission on Mental Health and COVID-19 (Trianni and e Souza, 2023) identified this fragmentation as one of the main reasons for pandemic recovery in the Americas. The Commission recommended not to continue to treat mental health as a separate, always under-resourced system but to integrate mental health into primary and community care instead. These conditions are not incidental. Examining students already receiving psychiatric care within this specific context tests whether the dual-continuum model retains explanatory power outside the high-income, English-speaking settings where it originated—in a health system where accessing a clinician constitutes an achievement in itself.

To our knowledge, no qualitative study has integrated these four conceptual frameworks—the dual-continuum model, ecological systems theory, professional identity formation, and psychological safety—within a sample already receiving psychiatric treatment, not merely screened for symptoms. Few have attempted this outside high-income, English-speaking contexts. We conducted semi-structured interviews with seven undergraduates attending a university-affiliated outpatient psychiatric clinic in Mexico City, reading each interview alongside that participant’s clinical record, not in isolation. Two questions guided the analysis: How do students under active psychiatric treatment narrate their emotional, psychological, and social well-being alongside their psychiatric symptoms? Which features of the university environment—across microsystem, mesosystem, exosystem, and macrosystem levels—render the coexistence of symptoms and flourishing more or less likely within individual accounts?

## 2 Materials and methods

### 2.1 Study design

We employed a qualitative, descriptive-interpretive design grounded in reflexive thematic analysis (Braun and Clarke, 2006, 2019). While participant recruitment was embedded within a broader quantitative survey administration week at the clinic, the present study reports exclusively on the qualitative data (semi-structured interviews triangulated with clinical records) without constituting a mixed-methods analysis. Fourteen deductive categories, derived from the eleven facets of Keyes' (2002) Mental Health Continuum, provided an initial analytical scaffold; all subsequent thematic structures emerged inductively from the dataset. Reporting adheres to the Consolidated Criteria for Reporting Qualitative Research (COREQ) checklist (Tong et al., 2007).

### 2.2 Setting and participants

The research setting was the outpatient mental health clinic of the Faculty of Medicine at Universidad Nacional Autónoma de México (UNAM) in Mexico City, a training facility providing psychiatric care to university students. During a designated census week, all students attending outpatient psychiatric consultations completed a well-being survey. That week was fixed in advance to fall outside examination periods, institutional holidays, and other events likely to distort typical clinic attendance or students’ reported well-being, so that the survey would approximate an ordinary week of care at this clinic. Those respondents whom the treating physician reported as clinically stable were subsequently invited to participate in semi-structured interviews. The final sample comprised students who were both deemed clinically stable by their treating physician and agreed to participate in an interview to explore their subjective experiences and meanings of well-being.

Eight students had initially agreed to be interviewed. In addition, one student who was a recent graduate and had already completed his coursework was excluded from the group. He had no academic obligations and was in transition to professional life (and therefore didn’t have time for the interview). This was done in order to provide a sample of undergraduate students who are already enrolled in the university with concurrent academic and clinical pressures.

The final sample consisted of seven students (six women, one man) aged 19–23 years old and enrolled in seven different degree programs at UNAM (Table 1). Because the interview subsample was restricted to survey respondents who volunteered for a subsequent conversation within that single recruitment week, *N* = 7 reflects a self-selected group bounded by a fixed time window, not a figure reached by expanding recruitment until no new themes emerged, so saturation, in the strict sense, was not the applicable criterion. Sample adequacy rests instead on information power (Malterud et al., 2016): the study’s narrow aim (students already under active psychiatric treatment, not merely screened for subclinical symptoms), its grounding in an established dual theoretical framework, and the triangulation of each interview with that participant’s clinical record together increase the information yield per case. Transferability, not statistical generalization, is the applicable standard, and is addressed further in Section 4.2.

**Table 1:** Sociodemographic and clinical characteristics of participants (*N* = 7).

Code	Sex	Age	Degree program	Academic progress	Principal psychiatric diagnosis	BDI-II	Notable psychosocial context
M20qfb	F	20	Pharmaceutical-Biological Chemistry	6th semester	MDD, moderate, with anxious features	39	Paternal bereavement (myocardial infarction, 1 year prior)
M22der	F	22	Law	8th semester	GAD + MDD, moderate + persistent depressive disorder	37	Suicide attempt (Oct. 2024); low perceived social support
M22cti	F	22	Earth Sciences	8th semester (thesis)	Persistent depressive disorder with intermittent MDD episodes	19	Concurrent work-study load; unresolved paternal bereavement
M19qui	F	19	Chemistry	not recorded	Not available in reviewed record (structured forms only)	18	Adolescent history of self-harm and disordered eating
M23soc	F	23	Sociology	10th of 8 (delayed)	GAD + persistent depressive disorder + ADHD	25	Financial barrier to treatment; peer household as primary support
M22mvz	F	22	Veterinary Medicine	≈5th–6th (estimated)	Not available in reviewed record (structured forms only)	36	First professional bereavement (patient death in clinical practice)
H23iq	M	23	Chemical Engineering	8th sem. + internship	MDD, moderate + GAD	Not recorded	Disciplinary proceeding; grandfather’s death witnessed directly

MDD, major depressive disorder; GAD, generalized anxiety disorder; ADHD, attention-deficit/hyperactivity disorder; BDI-II, Beck Depression Inventory-II (Beck et al., 1996), range 0–63. BDI-II not available for H23iq.

### 2.3 Data collection

A research psychiatrist (R. C. C.) conducted all interviews between March 28 and May 14, 2025. The interview guide was structured around Keyes’ three dimensions of well-being, yet administered with sufficient flexibility to allow participants to frame their lived experience instead of responding to a rigid checklist. The complete interview guide, in the original Spanish and in English translation, is available from the corresponding author upon reasonable request. Sessions were audio-recorded, transcribed verbatim, and de-identified prior to the onset of analysis.

### 2.4 Anonymization

Given the clinical sensitivity of the material and the small sample size, we enforced strict anonymization protocols. Each study participant was given an alphanumeric code based on their sex, age, and degree program abbreviation. For example, a 20-year-old woman in pharmaceutical-biological chemistry was assigned the alphanumeric code M20qfb. There are no personal identifiers of the research participants as they are not included in the analytic data at all.

### 2.5 Clinical data integration

Sociodemographic and clinical details were extracted from each participant’s outpatient psychiatric record. Extracted variables included the presenting complaint, current diagnosis, and relevant psychosocial history (e.g., bereavement, interpersonal violence, self-harm, substance use). Where available, standardized instrument scores were also recorded: the Beck Depression Inventory-II (Beck et al., 1996), the Beck Hopelessness Scale (Beck et al., 1974), the Beck Anxiety Inventory (Beck et al., 1988), and the Difficulties in Emotion Regulation Scale (Gratz and Roemer, 2004). Instrument coverage within the clinical record was not uniform across participants, reflecting the naturalistic character of routine outpatient documentation and not a fixed research battery applied to the whole cohort. The BDI-II was the most consistently documented measure across the sample (six of seven records) and is therefore reported systematically in Table 1; the Beck Hopelessness Scale, the Beck Anxiety Inventory, and the DERS were available only for a subset of records and, where present, are incorporated narratively into the Results instead of being tabulated. These clinical data were not utilized to validate or invalidate the participants’ self-reports. Instead, they served to contextualize the narrative accounts, operating on the premise that clinical records and interview discourse illuminate complementary dimensions of the same psychological reality.

### 2.6 Data analysis

Analysis followed Braun and Clarke's (2019) reflexive thematic analysis, understood as an active, iterative, and recursive process of meaning-making and not a mechanical coding procedure. The first and second authors engaged collaboratively with the dataset, reading and re-reading transcripts, generating initial codes, and developing candidate themes through sustained dialogue. Two members of the research team (the first and second authors) independently open-coded the first three transcripts, then met to compare coding decisions and consolidate a working coding framework; this framework was treated as a living document, revised throughout analysis as new transcripts were incorporated instead of being fixed in advance as a closed codebook. The remaining transcripts were then coded collaboratively against this evolving framework. Instead of seeking inter-coder agreement, the team engaged in reflexive discussion to surface divergent interpretations, treating these as analytically productive and not as ‘drift’ to be corrected (Braun and Clarke, 2019, 2021). The fourteen *a priori* Keyes categories served as an initial scaffold, but were continuously revisited as inductively generated subcategories and cross-cutting themes emerged. Quotations are presented in Spanish—the language in which participants actually spoke—accompanied by an English translation. This dual presentation preserves the original semantic and emotional texture, acknowledging that qualitative testimony loses nuance when translated without the source text at hand (Van Nes et al., 2010).

### 2.7 Ethical considerations

The study protocol was reviewed and approved by the Research Ethics Committee of the Faculty of Medicine, Universidad Nacional Autónoma de México (UNAM), under protocol number FM/DI/019/2026. Conducted in strict accordance with the Declaration of Helsinki, the study required all participants to provide written informed consent prior to enrollment. This consent explicitly covered the anonymized use of clinical record information for research purposes. Confidentiality was rigorously maintained throughout data collection, analysis, and reporting via the alphanumeric coding scheme detailed above. The informed consent form explicitly stated that de-identified sociodemographic and clinical information, including degree program, approximate age, academic progress, and diagnostic category, would be reported in aggregate or tabulated form for scientific dissemination. Participants were informed that this level of detail, despite the alphanumeric coding scheme, carries a residual risk of identification by individuals familiar with their immediate academic environment, and consented to this risk explicitly.

### 2.8 Researcher positionality and reflexivity

The psychiatrist (R. C. C.) who conducted all interviews, does not belong to the clinical care team treating patients at this specific outpatient clinic. This structural separation was fundamental to the study’s ethical design. It precluded any direct therapeutic power dynamic or conflict of interest between the interviewer and the participants. Because students were not interviewed by their own treating physicians, the risk of coercion or social-desirability bias was substantially mitigated. This professional distance likely facilitated more candid disclosures regarding institutional conflicts, treatment dissatisfaction, or personal struggles. Throughout the analysis, the research team maintained a reflexive stance, documenting how clinical training might shape the interpretation of narrative data, while acknowledging that the absence of a direct provider-patient relationship strengthened the ecological validity of the participants’ self-reports. Several concrete aspects of this reflexive stance deserve to be named explicitly, not simply assumed. The interviewer (R. C. C.) was a comparatively young male psychiatrist, a Colombian national interviewing Mexican undergraduates within a specifically Mexican clinical and institutional setting, while six of the seven participants were women. The narrow age gap likely reduced the generational distance that might otherwise have inhibited disclosure, even as the differences in gender and national origin persisted. Interviewer sex has been shown to shape both what participants disclose and how candidly they do so, though the literature does not converge on a single direction for this effect across topics and respondents (Broom et al., 2009; Verge, 2009); in this study, that configuration may have attenuated some participants’ willingness to describe experiences of harassment by male peers, while plausibly having facilitated H23iq’s account of perceived gender-based institutional treatment, a topic he may have addressed differently with a female interviewer sharing his own national context. All authors, in turn, are affiliated with the same university whose disciplinary and clinical structures are partly the object of this study; we treated that institutional proximity as an analytic resource and not a source to be neutralized, in line with reflexive accounts of positionality that regard the researcher’s standpoint as constitutive of the analysis, not external to it (Berger, 2015). Beyond gender, generation, and national origin, the situated, co-constructed character of the interview encounter is itself known to shape how participants express identity, independently of any single interviewer attribute (Escobar et al., 2018). This is part of why the interview format was semi-structured and conversational, not standardized: departing from a fixed script allowed R. C. C. to probe emergent narratives with a depth a rigid checklist forecloses, and to intervene actively with explanations, clarifications, or situational reframing whenever a participant’s understanding of a question appeared to diverge from its intended sense. Far from inflating interviewer variance or distorting responses, this conversational flexibility is associated in the survey-methodology literature with reduced measurement error, precisely because negotiating shared meaning in real time helps respondents map general concepts onto their own lived experience (Schober and Conrad, 1997). R. C. C.’s training as a psychiatrist further equipped him with interviewing strategies oriented toward eliciting detailed clinical narrative while limiting the imprint of his own presence on what participants chose to disclose. Finally, this reflexive vigilance was not left to a single person: as described in Section 2.6, coding and interpretation proceeded collaboratively between two members of the research team, specifically to surface and correct for interpretive bias that any one analyst, including the interviewer himself, might introduce.

## 3 Results

### 3.1 Participant characteristics

The final sample comprised seven undergraduate students (six women, one man) aged 19–23 years (M = 21.7), enrolled across seven distinct degree programs and ranging from the 6th to the 10th semester of study. All participants were under active psychiatric treatment at the time of the interview. Principal diagnoses spanned major depressive disorder, generalized anxiety disorder, persistent depressive disorder, and attention-deficit/hyperactivity disorder, frequently co-occurring within the same individual. Table 1 summarizes the sociodemographic and clinical characteristics of the cohort.

### 3.2 Emotional well-being: positive affect, satisfaction, and interest

#### 3.2.1 Happiness as connection, not achievement

Across the sample, positive affect rarely attached to academic performance metrics; instead, it emerged from the co-presence of peers sharing similar interests. M19qui summarized this as “el poder convivir con otras personas que tenían los intereses en común que yo tengo” [“being able to share time with other people who had the same interests I do”]. Similarly, M20qfb described “compartirlo con una persona que está viviendo esa experiencia contigo… se siente como un abrazo” [“sharing it with someone who is living that experience with you… it feels like a hug”], while M22mvz located happiness within professional practice itself [“cuando veo a mis pacientes bien” (“when I see my patients doing well”)]. In this context, positive affect appeared mediated by relational microsystems—peers, clinical practice, communities of interest—and not by objective performance indicators. This aligns with an ecological reading wherein happiness is generated at the interface between the student and their inhabited settings, not solely within the individual.

#### 3.2.2 Satisfaction as the closure of an effort cycle

Unlike happiness, satisfaction was consistently conceptualized as a *post hoc* state contingent upon the closure of an academic demand cycle: the end of a semester, passing an examination, or resolving a pending obligation. H23iq drew a notable distinction between present and retrospective satisfaction: “actualmente puedo decir que me estoy sintiendo más satisfecho que antes. Sin embargo, no puedo considerar que durante la carrera me he sentido… satisfecho” [“right now I can say I am feeling more satisfied than before. However, I cannot say that throughout my degree I have felt… satisfied”]. This indicates that the trajectory of well-being is neither linear nor stable, consistent with the dual-continuum premise that a single individual can oscillate between flourishing and languishing across different points of their formative trajectory.

#### 3.2.3 Vital interest as a motivational hinge

Interest emerged as the emotional category with the greatest explanatory reach into the other two dimensions. When interest declined, participants described a cascading disengagement. M22der noted, “no le encuentro un sentido a la vida… prefiero estudiar por mí misma… a venir a la escuela” [“I don’t find meaning in life… I would rather study on my own… than come to school”], while H23iq stated, “cuando pierdo el interés… dejo de entrar a clases, dejo de hacer tareas, dejo de hacer mi vida académica funcional” [“when I lose interest… I stop going to class, I stop doing homework, I stop functioning academically”].

The clinical record for M22der situates this account within an active diagnosis of generalized anxiety disorder, moderate major depressive disorder, and persistent depressive disorder, alongside a Beck Depression Inventory-II score of 37 and a suicide attempt by medication overdose 8 months prior to the interview. Read alongside this record, her account of vocational disengagement illustrates the coexistence between categorical illness and dimensional languishing predicted by the dual-continuum model. The narrative and the clinical data do not compete; they illuminate complementary dimensions of the same lived experience, capturing a depth that categorical nosology alone fails to register.

### 3.3 Psychological well-being: autonomy, self-acceptance, self-management, purpose, growth, relationships

#### 3.3.1 Autonomy self-limited by external validation

A consistent pattern emerged wherein participants conceptualized autonomy not as the absence of external constraint, but as a self-imposed deference to external validation. M22der explained, “soy muy indecisa y… le doy más importancia a las opiniones de los demás para tomar una decisión y no me enfoco en lo que yo quiero” [“I am very indecisive and… I give more weight to other people’s opinions when making a decision, instead of focusing on what I want”]. M19qui echoed this: “yo misma me limito a, a veces no a hacer lo que quiero, sino con lo que está mejor” [“I limit myself, sometimes not doing what I want but what seems better”]. This qualifies Keyes’ definition of autonomy as self-determination and resistance to social pressure. Within this population, psychological autonomy appears in active tension with internalized performance norms, a dynamic that warrants further investigation against the literature on perfectionism in high-demand degree programs.

#### 3.3.2 Self-acceptance traversed by grief and loss

Self-acceptance manifested not as a static trait but as a dynamic process catalyzed by disruptive life events. M20qfb linked her shift in self-perception directly to her father’s death: “lo que llevó a eso fue la muerte de mi padre el año pasado, lo cual me llevó a reprobar materias” [“what led to that was my father’s death last year, which led me to fail courses”]. H23iq narrated an identity reconstruction following his grandfather’s death and a romantic rupture: “he aprendido que al final del día uno está solo… nadie te va a sacar del hoyo en el que tú te sientas” [“I have learned that at the end of the day you are alone… no one is going to pull you out of the hole you have sunk into”].

The clinical record provides precise chronology for these narratives. M20qfb’s father died of an acute myocardial infarction in March 2024, approximately 1 year before her pre-assessment. Her Beck scores (Depression 39, Anxiety 43) place her in the severe range, accompanied by suicidal ideation without a structured plan. H23iq’s record adds further context: within the same 18 months as his grandfather’s bereavement, he underwent a university disciplinary process stemming from an accusation of threatening a former partner, was publicly exposed on a student platform, and was socially ostracized by his cohort. The co-occurrence of acute grief with an institutional conflict of this magnitude demonstrates that the personal growth participants narrate does not occur in a vacuum; it unfolds in active tension with the microsystem.

#### 3.3.3 Self-management as a containment strategy under overload

Self-management (organization, discipline, responsibility) was almost always described in an instrumental register of coping, not of growth per se: “organizar mi día, lo que tengo que hacer cada día” [“organizing my day, what I have to do each day”] (M22der); “calendarizar y gestionar… prever o planear” [“scheduling and managing… anticipating or planning”] (M23soc). M22mvz linked it explicitly to disciplined persistence even without intrinsic motivation [“aunque no te guste, pues hacerlo” (“even if you do not like it, you just do it”)]. In the clinical-university microsystem, self-management appeared to function as a compensatory mechanism against academic load, not as a well-being facet experienced in its own right.

#### 3.3.4 Purpose in life: from professional ambition to emotional equilibrium

Several narratives revealed an ongoing reformulation of life purpose, shifting from initially ambitious professional goals toward objectives centered on emotional regulation and inner peace. M20qfb noted, “antes… decía… voy a llegar a ser una investigadora… pero ahorita… mi principal propósito en la vida es entenderme” [“before… I used to say… I am going to become a researcher… but right now… my main purpose in life is to understand myself”]. Similarly, M22der stated, “mi propósito en la vida ahorita es tener paz y tranquilidad. Estoy muy inestable emocionalmente” [“my purpose in life right now is to have peace and calm. I am very emotionally unstable”]. This shift from achievement-oriented purpose toward affective stabilization coincided temporally with each participant’s period of greatest reported distress, suggesting that purpose in life reorganizes dynamically in response to perceived distress load.

#### 3.3.5 Challenges and personal growth: the microsystem as generator of adversity and learning

Reported challenges were heterogeneous but converged in being situated within concrete microsystems of university life: harassment by a fellow social-service intern (M19qui); emotional exhaustion from an unresolved grief process (M20qfb); commuting difficulties and their relationship to attrition [“ahora entiendo por qué muchos dejan la universidad por lo mismo del tiempo de transporte… o no les alcanza el dinero” (“now I understand why many people drop out of university for the same reason, commuting time… or they don’t have enough money”), M22der]; the onset of paid work alongside coursework (M22cti); romantic rupture and constant partner criticism (H23iq); a first patient death during clinical practice (M22mvz); and living alone with no immediate support network (M23soc). Two presenting complaints, read verbatim, condense this ecological tension particularly well. M22cti’s read: “en los últimos meses… he tenido momentos donde siento que ya no quiero hacer las cosas que tengo que hacer y me abruma mucho el ritmo de trabajo y escuela que estoy llevando, además de que el ambiente en casa es complicado” [“in recent months… there have been moments when I feel I no longer want to do the things I have to do, and I feel overwhelmed by the pace of work and school I’m keeping up, on top of a complicated home environment”], work, school, and home named in the same sentence as simultaneous sources of demand. M23soc’s presenting complaint was explicitly economic: “he llevado ya tratamiento psiquiátrico y no tengo la solvencia económica para pagar medicamentos y la terapia” [“I have already been in psychiatric treatment and I do not have the financial means to pay for medication and therapy”], a treatment-access barrier that is, in itself, an exosystemic datum (the mental health services market, student economic precarity) conditioning the clinical course observed.

#### 3.3.6 Close positive relationships as a buffering cushion

Almost without exception, friendship and family relationships were described as the most frequently cited protective resource, operating even without active intervention [“no hace falta que haya conversaciones, sino… la vibra que siento en los círculos… en mi casa, con mis amigos, en pareja” (“there does not need to be conversation, just… the vibe I feel within these circles… at home, with my friends, with my partner”), M22cti]. Notably, M23soc described her group of roommates as “un muy buen sustituto de familia” [“a very good substitute for family”], evidence of a reconfiguration of primary support networks during the transition to university life, a mesosystem phenomenon in Bronfenbrenner’s terms. Her clinical record makes clear why this substitution was necessary: M23soc describes a nonexistent relationship with her father and an only “so-so” relationship with her mother, while cohabiting, for some time, with four roommates (three women and one trans woman) and two cats, the family-of-origin mesosystem recedes precisely as the peer mesosystem expands to occupy its functional place.

### 3.4 Social well-being: acceptance, coherence, growth, contribution, integration

#### 3.4.1 Social trust: distrust learned through experience, not trait

Social acceptance and interpersonal trust were narrated in a markedly defensive register: “me cuesta mucho trabajo confiar en las personas” [“it is very hard for me to trust people”] (M22der); “soy bastante desconfiada con la gente por experiencias que me ha dado la vida” [“I am quite distrustful of people because of experiences life has given me”] (M19qui); “conforme van pasando los años me cierro más” [“as the years go by, I close myself off more”] (M22mvz). In no case was this presented as a stable personality trait; it was instead narrated as the outcome of specific experiences of betrayal or disappointment, many of them within the university environment itself.

#### 3.4.2 Social coherence: a competitive, individualistic system, with a gender-specific nuance

Several participants described their faculty’s culture as individualistic and competitive [“ambiente bastante raro entre compañeros, son muy individualistas” (“a rather strange atmosphere among classmates, they are very individualistic”), M19qui; “todos quieren tener… una pelea de poder… no quiero compartirlo con los demás” (“everyone wants… a power struggle… I don’t want to share it with the others”), M22der]. H23iq introduced a distinct and potentially important nuance for institutional psychological safety: he perceived institutional norms as gender-inequitable [“si eres hombre se te devalúa, no tienes credibilidad… nada más se le escuche a la mujer y no se le dé el beneficio de la duda al hombre” (“if you are a man you get devalued, you have no credibility… only the woman gets listened to, and the man is not given the benefit of the doubt”)]. As the sample’s only male participant, H23iq was also the only one to frame institutional distress in terms of perceived gender-based inequity. This single account cannot establish a systemic pattern, but it points to specific institutional adjudicative processes, not a diffuse campus climate, as the source of compromised psychological safety, a distinction that future research with more male participants should examine directly.

#### 3.4.3 Contribution and emerging professional identity

Social contribution was articulated almost entirely through participants’ field of study as a vehicle of social purpose, pharmaceutical chemistry [“mi carrera… tiene un impacto muy grande” (“my degree… has a very large impact”) on health, M20qfb]; law [“puede ayudar a mejorar esa situación” (“it can help improve that situation”) of insecurity, M22der]; earth sciences (M22cti); veterinary medicine [“sin la veterinaria… estaríamos llenos de pandemias” (“without veterinary medicine… we would be full of pandemics”), M22mvz]. Contribution did not appear as an abstract value; it fused with the disciplinary identity under formation, the student does not merely “learn a profession” but begins to narrate the self through it, consistent with the professional identity formation literature’s account of health-sciences and science-based training as identity-reorganizing well before graduation (Cruess et al., 2014, 2016).

#### 3.4.4 Social integration: belonging as a polarized experience

This was the social axis with the greatest contrast between cases, and therefore the richest for illustrating the continuum, not the dichotomy, of social mental health. At one extreme, M22mvz: “siempre me he sentido aceptada en mi facultad… te sientes incluido, cuidado por todos” [“I have always felt accepted in my faculty… you feel included, looked after by everyone”]. At the opposite extreme, H23iq: “mi pertenencia a la universidad es nula… fue una institución que a mí me dio la espalda cuando yo necesitaba su ayuda… la gente te juzga con la mirada… te aíslan en grupos… te hacen sentir una persona marginada” [“my sense of belonging to the university is null… it was an institution that turned its back on me when I needed its help… people judge you with their eyes… they isolate you within groups… they make you feel like a marginalized person”], an account that borders on the language of institutional ostracism. Between these poles, M19qui contributed the most interesting temporal nuance: she described solid overall belonging while acknowledging recent episodes of disconnection tied to vocational questioning [“estos últimos meses no me he sentido parte de… pensar si realmente lo que estoy estudiando me gusta” (“these last few months I haven’t felt part of it… wondering whether I really like what I’m studying”)]. This last account is, in our reading, the clearest example in the corpus of bicontinuous fluctuation within a single subject over a short time window.

### 3.5 Cross-cutting themes

Beyond the fourteen a *priori* facets, five patterns kept surfacing across categories, and these are what should organize the Discussion below instead of a facet-by-facet recap.

**Theme A.** Complete mental health as coexistence, not absence of symptoms. This constitutes the most robust finding in the corpus. Well-being and distress did not alternate within the interviews; nearly every participant described a significant event of suffering—bereavement, harassment, a patient’s death, financial precarity—concurrently with an active indicator of flourishing: purpose, interest, or a sustaining relationship. The available BDI-II scores ranged from 18 to 39 (mild to severe), and no participant’s narrative reduced to symptoms alone. The clinical metrics and the discourse articulate the same reality independently.

**Theme B.** The university as an ambivalent microsystem. It gave meaning, contribution, a professional identity in the making, and in the same institution, sometimes the same week, sometimes the same relationship, it generated threat: competition, individualism, transportation costs nobody accounts for. This is not two different environments. It is one, and which face it shows seems to depend on which relational subsystem happens to be active at the time.

**Theme C.** Interest as the hinge. Vital interest never stayed inside Keyes’ emotional dimension, where the model technically places it. It pulled self-management, purpose, and social integration along with it, and when it dropped, the others tended to follow. That looks less like a facet and more like a lever.

**Theme D.** Peer networks stepping into family’s place. More than one participant had, without quite naming it that way, replaced or supplemented a family-of-origin network with roommates or a clinical-practice cohort. Bronfenbrenner would call this a mesosystem shift; the participants called it, more simply, finding people who felt like home.

**Theme E.** Institutional psychological safety and disciplinary processes: a critical case illustration.

The single male participant in the sample described an inequity the six women did not, anchored not in a vague impression but in an actual disciplinary episode involving a tribunal, a ruling, and public exposure. While *N* = 1 precludes broad generalizability, this case provides a strikingly clean illustration of how specific institutional processes can compromise a student’s sense of safety, warranting serious analytical consideration (see Figure 1; Table 2).

**Figure 1:**
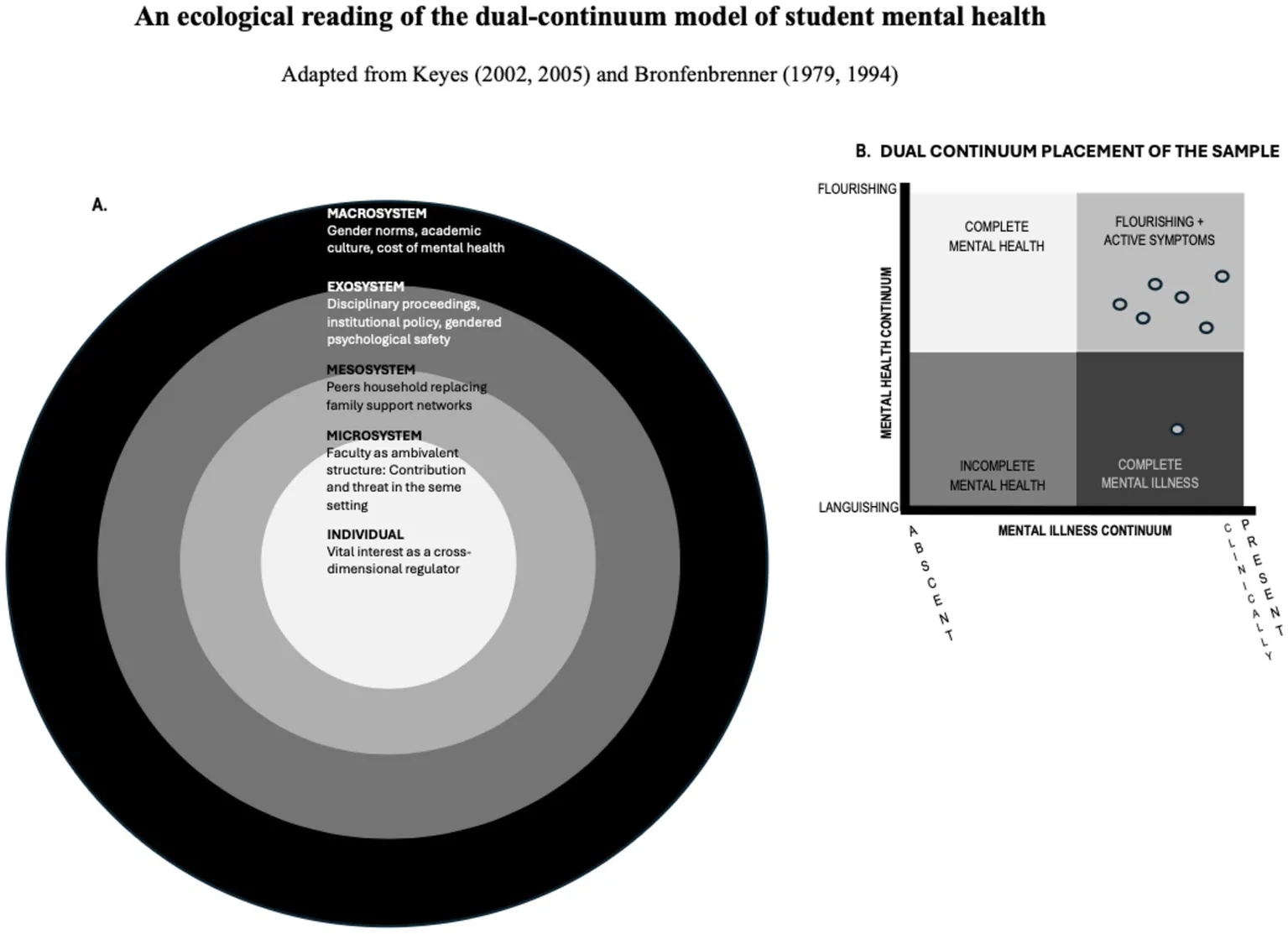
An ecological reading of the dual-continuum model of student mental health, adapted from Keyes (2002, 2005) and Bronfenbrenner (1979, 1994). The rings **(A)** place the five cross-cutting themes at the ecological level where each mostly plays out; the inset plot places **(B)** each participant along Keyes’ two continua, and six of the seven land in the “flourishing with active symptoms” quadrant.

**Table 2:** Representative quotations by theme.

Theme	Original quotation (Spanish)	English translation	Code
Happiness as connection	“el poder convivir con otras personas que tenían los intereses en común que yo tengo”	“being able to share time with other people who had the same interests I do”	M19qui
Interest as hinge	“cuando pierdo el interés… dejo de entrar a clases, dejo de hacer tareas, dejo de hacer mi vida académica funcional”	“when I lose interest… I stop going to class, I stop doing homework, I stop functioning academically”	H23iq
Self-acceptance/grief	“lo que llevó a eso fue la muerte de mi padre el año pasado, lo cual me llevó a reprobar materias”	“what led to that was my father’s death last year, which led me to fail courses”	M20qfb
Purpose reorganized	“mi propósito en la vida ahorita es tener paz y tranquilidad. Estoy muy inestable emocionalmente”	“my purpose in life right now is to have peace and calm. I am very emotionally unstable”	M22der
Peer network as family	“un muy buen sustituto de familia”	“a very good substitute for family”	M23soc
Contribution/identity	“sin la veterinaria… estaríamos llenos de pandemias”	“without veterinary medicine… we would be full of pandemics”	M22mvz
Institutional (un)safety	“mi pertenencia a la universidad es nula… fue una institución que a mí me dio la espalda cuando yo necesitaba su ayuda”	“my sense of belonging to the university is null… it was an institution that turned its back on me when I needed its help”	H23iq
Bicontinuous fluctuation	“estos últimos meses no me he sentido parte de… pensar si realmente lo que estoy estudiando me gusta”	“these last few months I have not felt part of it… wondering whether I really like what I’m studying”	M19qui

## 4 Discussion

A central finding of this study is that flourishing and clinically significant psychiatric symptoms co-occurred within participants’ narratives, not merely alternated. For the majority of the sample, these dimensions were temporally and experientially inseparable: accounts of active psychological distress (e.g., unresolved grief, institutional ostracism, disordered eating, a recent suicide attempt) were articulated concurrently with indicators of purpose, vital interest, and social contribution. This empirical pattern aligns with the upper-right quadrant of Keyes’ (2005) complete state model of mental health, demonstrating that a diagnosable disorder can coexist with high levels of emotional, psychological, and social well-being. Contrary to assumptions that clinical diagnosis inherently implies languishing, our data illustrate a complex, simultaneous manifestation of both continua. While most evidence supporting this quadrant derives from community samples screened for symptoms (Keyes, 2005, 2007; Lamers et al., 2011), the present findings extend this observation to a clinical population. Beck Depression Inventory-II scores in this cohort ranged from 18 to 39, indicating mild to severe depressive symptomatology (Beck et al., 1996); nevertheless, no participant’s narrative reduced exclusively to pathological descriptors.

These phenomenological accounts do not exist in a vacuum; they are firmly anchored within specific ecological contexts. For nearly every participant, the university functioned as an ambivalent microsystem. The same clinical rotation that exposed M22mvz to her first professional bereavement—a patient’s death—was simultaneously the setting where she reported the strongest sense of belonging within the entire cohort. Bronfenbrenner (1979, 1994) would argue that this is not a contradiction; the valence of a microsystem is never static, but rather contingent upon which relational subsystem is currently activated. Consequently, contribution and disciplinary identity emerged as the most robust bridges between the psychological and social dimensions. Students across pharmaceutical chemistry, law, earth sciences, and veterinary medicine located their sense of social purpose directly within the profession they were actively constructing, not in abstract civic ideals. This observation echoes the premise that training in high-stakes, vocation-driven disciplines (such as health sciences, law, or engineering) reorganizes a student’s self-concept around disciplinary values well before graduation (Cruess et al., 2014, 2016). For several participants, this emerging professional identity operated not merely as a byproduct of effective coping, but as an active stabilizing resource in its own right.

The narrative of H23iq, the sole male participant in the sample, presented a qualitatively distinct pattern of institutional distress. His account of ostracism was not a diffuse mood or a vague impression; it traced directly to a formal disciplinary proceeding that escalated to the university tribunal, required 15 months to resolve in his favor, and involved public exposure on a student platform. Viewed in isolation, his assertion that “if you are a man you get devalued” represents a single case that precludes generalization. However, interpreted through the lens of psychological safety literature, it provides a striking illustration of how specific institutional adjudicative processes, and not a generalized campus “climate,” can severely compromise a student’s perception of environmental safety (Dong et al., 2025; Edmondson, 1999). Research on psychological safety in health-professions education has predominantly focused on ward rounds, simulation labs, and supervisory relationships (Dong et al., 2025), leaving disciplinary and adjudicative mechanisms largely unexamined as distinct threats. This case suggests that institutional justice—specifically, how accusations are processed and managed—warrants examination as a critical mesosystem-exosystem interface.

Across the cohort, vital interest functioned less as an isolated emotional state and more as a structural hinge. When interest declined, engagement with coursework, self-management strategies, and peer contact tended to deteriorate concurrently; when it remained intact, it sustained purpose and social contribution even under substantial clinical weight. Keyes (2002) previously noted that the eleven facets of his model correlate and do not operate in isolation; the present data refine this observation by suggesting a directional hypothesis—that interest may exert a driving influence on other well-being dimensions and not merely correlate with them. This proposition remains a hypothesis, as a cross-sectional interview design is inherently unsuited to confirm causal directionality; longitudinal or mixed-methods approaches are required for such validation. Finally, multiple participants had reconfigured their primary support networks around peers—roommates or clinical-practice cohorts—and not around their families of origin. Bronfenbrenner (1979) would classify this as a mesosystemic adaptation, and in at least one instance, this shift was directly traceable to a documented rupture with the family of origin recorded in the clinical file.

### 4.1 Clinical and educational implications

Relying exclusively on symptom severity measures obscures critical information necessary for effective intervention: namely, whether a student’s sense of purpose, professional identity, and belonging remains intact, is actively eroding, or has already dissipated. Integrating brief flourishing assessments alongside standard symptom screening in university or health-professions clinics could identify students whose languishing precedes overt symptomatology, or whose functional impairment persists despite symptom remission. Clinically, this suggests that a sudden decline in vital interest may serve as an early, observable marker of languishing, preceding or exacerbating the worsening of categorical symptoms. Routine clinical assessments in university settings should therefore probe for the presence of ‘interest’ and ‘purpose’ with the same rigor applied to symptom checklists, as their absence may signal an impending crisis of meaning, not merely a transient mood fluctuation. In practice, this could take the form of administering a brief instrument such as the Mental Health Continuum-Short Form (Lamers et al., 2011) at intake and at routine follow-up, alongside existing symptom measures, so that flourishing is tracked as its own trajectory and not simply inferred from symptom remission. Embedding this within intake and follow-up protocols already in place, instead of adding it as a separate assessment, would limit the additional burden on clinic staff and on students already navigating frequent evaluation. At the institutional level, psychological safety must transition from being conceptualized as an ambient feature of “campus climate” to being understood as a tangible outcome produced or destroyed by specific procedural mechanisms. Disciplinary proceedings, complaint resolution protocols, and policies regarding public exposure require the same rigorous procedural scrutiny currently applied to clinical processes. Additionally, because several participants located both their most profound distress and their clearest sense of meaning within their emerging profession, identity-formation support should be embedded directly within student mental health services, not relegated to curricular design committees.

### 4.2 Limitations

There are some limitations in this study. First, the study is small and conducted at a single UNAM-affiliated clinic in Mexico City, limiting the transferability of findings to other institutions, disciplines, or cultural contexts. Second, the sample was heavily skewed toward women (six of seven); while the single male account is analytically rich, it cannot be generalized. Future research should deliberately recruit for gender balance, and in particular for a larger subsample of male students, to establish whether the perceived gender-based asymmetry in institutional treatment described by H23iq reflects an idiosyncratic account or a broader pattern that would warrant institutional attention in its own right. Third, the cross-sectional, single-interview design precludes the establishment of causal directionality—whether professional identity shapes well-being or vice versa—and prevents tracking how the coexistence of flourishing and symptoms evolves across an academic trajectory. Fourth, clinical records were not uniformly comprehensive: five participants possessed detailed narrative psychiatric histories, whereas two (M19qui and, to a lesser extent, M22mvz) were represented primarily by structured pre-assessment forms, yielding thinner contextual data for those specific cases. Fifth, although the interviewing psychiatrist did not belong to the participants’ direct clinical care team, the institutional proximity may have introduced some degree of social-desirability bias. However, the explicit disclosures of self-harm, suicidal ideation, and institutional conflict suggest that any such effect was minimal. Finally, all quotations were translated from Spanish to English. While the original text is provided alongside the translation to mitigate semantic loss (van Nes et al., 2010), some idiomatic and emotional nuance is inevitably attenuated in the process.

## 5 Conclusion

This qualitative investigation demonstrates that university students undergoing active psychiatric treatment for moderate to severe depressive and anxious symptomatology consistently articulate robust indicators of flourishing—purpose, vital interest, professional identity, and social contribution—simultaneously with their clinical distress. Viewed through the integrated framework of Keyes’ dual-continuum model and Bronfenbrenner’s ecological systems theory, this simultaneous manifestation is neither incidental nor strictly intrapsychic. Rather, it appears closely tied to distinct features of the university training environment, consistent with, though not proof of, the ecological and dual-continuum frameworks that guided this analysis. These include the ambivalent nature of the academic microsystem, the functional reconfiguration of primary support networks toward peer groups, and the presence or absence of institutional psychological safety during disciplinary processes. For health-professions education, where professional identity formation constitutes a core curricular objective, these findings delineate a clear operational distinction: supporting student flourishing and treating psychiatric symptoms are related, yet fundamentally non-interchangeable, clinical and educational tasks. Institutions committed to safeguarding student mental health must therefore evaluate the ecological and identity-related conditions that enable flourishing with the same rigor applied to symptom remission.

## Data Availability

The raw data supporting the conclusions of this article will be made available by the authors, without undue reservation.
